# Genome Sequencing of *Ralstonia solanacearum* Race 4, Biovar 4, and Phylotype I, Strain YC45, Isolated from *Rhizoma kaempferiae* in Southern China

**DOI:** 10.1128/genomeA.01110-15

**Published:** 2015-10-01

**Authors:** Xiaoman She, Yafei Tang, Zifu He, Guobing Lan

**Affiliations:** a Plant Protection Research Institute Guangdong Academy of Agricultural Sciences, Guangzhou, China; b Guangdong Provincial Key Laboratory of High Technology for Plant Protection, Guangzhou, China

## Abstract

*Ralstonia solanacearum* is an important phytopathogen that attacks over 400 plant species, including *Zingiberaceae* plants. Here, we report the complete genome sequence of strain YC45, which was isolated from *Rhizoma kaempferiae* in southern China.

## GENOME ANNOUNCEMENT

*Ralstonia solanacearum* is an important phytopathogen that attacks over 400 plant species in tropical, subtropical, and some temperate areas of the world (1, 2). The bacterium has been subclassified into five biovars according to its ability to utilize carbohydrates, and into five races based on its host range. Further, molecular studies have revealed high diversity among strains of *R. solanacearum*, and it is therefore considered a species complex. In southern China, *R. solanacearum* has infected more than 15 plants and caused bacterial wilt diseases, especially in tomato, eggplant, pepper, ginger, *Rhizoma kaempferiae*, and so on. Strain YC45 was isolated from a diseased *Rhizoma kaempferiae* plant exhibiting typical bacterial wilt symptoms in Yangchun city, Guangdong province, China, and was confirmed to be the pathogen of *Rhizoma kaempferiae* bacterial wilt (3). YC45 belonged to race 4, biovar 4 and phylotype I. The complete genome of YC45 is reported here.

The genome sequence of YC45 was determined using the PacBio RS and Illumina HiSeq 2000 platforms. The final sequence assembly was carried out using HGAP version 3. The PacBio *de novo* assembler was optimized for speed (4), and putative protein-coding sequences (CDSs) were predicted by Glimmer (5) and GeneMark (6). Functional annotation was based on BLASTp searches against the KEGG and NR databases. tRNA genes were directly predicted with tRNAscan-SE (7). Clusters of Orthologous Groups of proteins (COG) assignments were performed by RPS-BLAST using the NCBI Conserved Domains Database (CDD) library.

In total, a complete chromosome sequence of 3,724,487 bp and a complete megaplasmid sequence of 2,008,422 bp of YC45 were generated, resulting in a 5.73-Mb YC45 genome, which is ~50 kb larger than the average size (5.68 Mb) of the sequenced *R. solanacearum* strains. The average GC content of the chromosome is 67.193%, while that of the megaplasmid is 66.896%. The entire genome contains 4,621 CDSs, 2 complete rRNA loci, and 46 tRNA genes. Of the 4,621 CDSs, 3,687 proteins can be assigned to COG families (8). A total of 2,663 proteins had KEGG orthologs, and these proteins were involved in 109 pathways. Among the 4,621 CDSs, 3,987 proteins were assigned biological functions; 1,025 CDSs are homologous to hypothetical proteins of unknown function. The remaining 137 hypothetical proteins had no match to any known proteins in the databases. Compared with the deposited *R. solanacearum* genomes in GenBank, YC45 was most closely related to phylotype I strain GMI 1000.

### Nucleotide sequence accession numbers. 

The *Ralstonia solanacearum* strain YC45 chromosome and megaplasmid sequences have been deposited in GenBank under the accession numbers CP011997 and CP011998, respectively.
